# Stiff-Elongated Balance of PLA-Based Polymer Blends

**DOI:** 10.3390/polym13244279

**Published:** 2021-12-07

**Authors:** Mónica Elvira Mendoza-Duarte, Iván Alziri Estrada-Moreno, Perla Elvia García-Casillas, Alejandro Vega-Rios

**Affiliations:** 1 Centro de Investigación en Materiales Avanzados, S.C., Av. Miguel de Cervantes #120, Chihuahua 31136, Mexico; monica.mendoza@cimav.edu.mx; 2 Instituto de Ingeniería y Tecnología, Universidad Autónoma de Ciudad Juárez, Chihuahua 32310, Mexico; 3 CONACyT-CIMAV, S.C., Av. Miguel de Cervantes #120, Chihuahua 31136, Mexico; ivan.estrada@cimav.edu.mx

**Keywords:** PLA, EVA, SMMA, polymer blends, mechanical properties balance, stiff polymer, elongated polymer

## Abstract

In this study, polymer blends with a mechanical property balance based on poly(lactic acid) (PLA), stiff polymer, and elongated polymer were developed. First, the binary blends PLA-elongated polymer [ethyl vinyl acetate (EVA) or polyethylene], or PLA-stiff polymer [polystyrene or poly(styrene-*co*-methyl methacrylate) (SMMA)] blends were studied using dynamic mechanic analysis (DMA) and analyzed using Minitab statistical software to determine the factors influencing the elongation or stiffness of the blends. Then, ternary blends such as elongation-poly(lactic acid)-stiff, were made from the binary blends that presented optimal performance. In addition, three blends [EVA–PLA–SMMA (EPS)] were elaborated by studying the mixing time (5, 15, and 15 min) and the added time of the SMMA (0, 0, and 10 min). Specifically, the mixing time for EPS 1, EPS 2, and EPS 3 is 5 min, 15 min, and 15 min (first EVA + PLA for 10 min, plus 5 min PLA-EVA and SMMA), respectively. Mechanical, thermal, rheological, and morphological properties of the blends were studied. According to DMA, the results show an increase in elongation at break (*ε*_b_) and do not decrease the elastic module of poly(lactic acid). Nevertheless, EPS 3 excels in all properties, with an *ε*_b_ of 67% and modulus of elasticity similar to PLA. SMMA has a significant role as a compatibilizing agent and improves PLA processability.

## 1. Introduction

Biodegradable polyesters have been studied extensively in various engineering fields, including medical, biomedical, and packaging [1]. In particular, poly(lactic acid) (PLA) thermoplastic has a tensile strength ranging from 50 MPa to 70 MPa and an elastic modulus equivalent to 4.0 GPa, depending on molecular weight and stereochemical composition. Additionally, PLA-based multicomponent polymer blends are raising interest in specific applications such as tissue engineering, pharmaceutical, sutures, and drug delivery [2,3]. Even though PLA has been extensively studied, there are still challenges to solving its intrinsic properties, including stiffness-toughness balance, improved processability and interfacial adhesion (polymer blends [4], composites [5], or hybrid composites [6]), and lastly enhanced heat resistance [7].

Moreover, the high brittleness (with an elongation at break, *ε*_b_, inferior to 10% [8]) is a typical problem involved in achieving a stiffness-toughness balance for a particular PLA application [7,9]. To overcome the mechanical limitations of PLA, diverse methods have been studied, such as copolymerization, grafting [1], blending with other polymers [10], the addition of plasticizers [2,11], and a combination of these strategies [12]. However, these pathways present limitations because they generally improve only one or various specific PLA properties (in the identical sense of direction). For example, plasticizers are well known to improve *ε*_b_ of the PLA; however, the elastic modulus decreases, i.e., its mechanical strength [8]. In addition, plasticizers can accelerate the degradation through the hydrolysis of PLA during the melt mixing process because they contain hydrophilic groups in their chemical structure [13].

Currently, there is research aimed at improving a stiffness-toughness balance utilizing the polymer blend method. For example, Han et al. [14] improved the compatibility of a PLA/poly (butylene adipate-*co*-butylene terephthalate) (PBAT) blend utilizing a chemical reaction of epoxidized soybean oil with terminal hydroxyl groups during the two phases. The obtained blends reported a significant increase in mechanical properties of tensile strength, *ε*_b_, and impact strength. Additionally, Huang et al. [15] reported the formation of PLA/modified polycaprolactone blends through electron-induced reactive processing without the addition of any chemical compatibilizers. They also reported a higher modulus (analyzed by DMA) and a positive modification on impact toughness. Likewise, Chen et al. [16] elaborated on a stable co-continuous morphology of PLA/PBAT (70/30) blends compatible with poly(styrene-*co*-glycidyl methacrylate) (SC) through reactive blending. They reported synergistically enhanced mechanical property, precise tensile strength, and specific ductility, with the synthesis of a compatibilizing agent between PDLA or PLLA and SC playing a significant role in the blend and morphology.

Additionally, the morphology achieved in PLA-based polymer blends is usually heterogeneous or homogeneous. The heterogeneous ones generally have mechanical properties superior to homogeneous ones [17], however, except when mixing with elastomers because phase separation occurs [18]. Nevertheless, some problems may appear as unstable mixtures, and consequently, the mechanical properties can be lost over time [19].

The importance and originality of this study is that it explores a balance of mechanical properties, particularly stiff-elongated, for PLA-based polymer blends. In addition, the PLA-based blend was manufactured from a selection of elongated polymer [ethyl vinyl acetate (EVA) or low-density polyethylene (LDPE)] and stiff polymer [polystyrene (PS) or poly(styrene-*co*-methyl methacrylate) (poly(S-*co*-MMA)], EVA being a copolymer with properties according to the composition of its two constituent homopolymers, polyethylene (PE) and poly(vinyl acetate). Recent studies of PLA-EVA binary blends report super toughness properties resulting in improved *ε*_b_, utilizing a compatibilizing agent (ethylene-methyl acylate-glycidyl methacrylate) [20], dicumyl peroxide (initiator and cross-linking) [21], and silica nanoparticles [21]. Likewise, PLA-LDPE blends offer benefits such as cost-performance and enhancement of the degradability of LDPE [22]. Ferri et al. [23] reported improved *ε*_b_ for PLA-biobased high-density PE blends; however, a compatibilizing agent (EVA) is needed in the blend. Quitadamo et al. [24] produced PLA-PE blends to balance the amount of bio-derived charge and mechanical properties when the blend contained equal amounts of PE and PLA. Additionally, they reported the addition of glycidyl methacrylate at 3% as a compatibilizing agent, improving the mechanical properties and the polymer affinity.

On the other hand, a few authors studied the properties of PLA–PS blends and reported values between the neat polymers [25,26,27]. As a result, PS could become a critical ingredient for developing various products, such as medical devices and packaging products, owing to clarity, dimensional stability, and adaptability to radiation sterilization [26,27]. Regarding PLA-poly (styrene-*co*-methyl methacrylate) (poly (S-*co*-MMA)) blends, Solorio and Vega [28] reported the melt blending of amorphous PLA with poly (S-*co*-MMA). They found that blends exhibit improved properties concerning amorphous PLA through mechanical and rheological characterization.

Herein, we report on PLA-based polymer blends with mechanical properties balance, specifically stiff-elongated. The study’s main aim is to increase elongation and preserve the PLA elastic module through melt blending. Consequently, there are two specific objectives of this study: 1—To develop a PLA-based polymer blend from the individual study of PLA-stiff polymer and PLA-elongated polymer blends; 2—To investigate the effect of the processing method on the mechanical properties balance of the ternary blend. In truth, there are few investigations concerning SMMA addition during mixing and its effect on the properties of the polymer blend (EVA or PE)_x_–PLA_y_–(poly (S–*co*–MMA) or PS)_z_.

## 2. Materials and Methods

### 2.1. Materials

Poly (l,d–lactide) PLA Ingeo 4060D, (8–10%) D [29], glass transition temperature *T_g_* = 55 °C–60 °C from NatureWorks LLC, USA. Low-density polyethylene (CERTENE^®^ LDPE 219A), ρ = 0.919 g/cm^3^, *MFI* = 2. Ethylene vinyl acetate (EVA) commercial name EVA 2810-A ATEVA CELANESE with 28% vinyl acetate, ρ = 0.949 g/cm^3^ and *MFI* = 6. Polystyrene (Sigma-Aldrich 430102) Mw-192,000, *MFI* 6.0–9.0. Poly(Styrene-*co*-methyl methacrylate), SMMA NAS^®^ 30, ρ = 1.090 g/cm^3^, *T_g_* = 103 °C, *MFI* = 2.2, from Ineos Styrolution Group GmbH, Germany. The styrene and methyl methacrylate content on Poly(S-*co*-MMA) ranged from (70 to 90)% and from (10 to 30)%, respectively.

### 2.2. Melt Blending

The polymer blends were prepared in a Brabender internal mixer (BB) (DDRV501, C.W. Brabender Instruments Inc., Hackensack, NJ, USA) at a temperature of 180 °C. Table 1 shows the formulations of manufactured polymer mixtures. To remove moisture that polymers might contain and prevent possible hydrolysis degradation during processing, pellets were dried at a temperature of 60 °C for 12 h utilizing an Isotemp model 281 vacuum oven.

### 2.3. Binary Blends Characterization

#### 2.3.1. Tensile Tests

Tensile tests were performed in a DMA RSA III from TA Instruments (New Castle, DE, USA), with a load cell of 3.5 kg. The applied crosshead speed was 50 mm/min. The tensile tests were conducted on rectangular samples with a gauge length section of 10 mm × 5 mm × 0.4 mm (*L* × *W* × *T*). In addition, three samples were tested for each blend. The study was conducted at a temperature of 37 °C.

#### 2.3.2. Statistical Analysis

To determine the variables significant for elongation at break (*ε*_b_) and elastic modulus (*E*) parameters, the statistical analysis of the samples was carried out considering a level of significance of α = 0.05. The statistical analysis is supported by a factorial regression table, Pareto graph of standardized effects, normal graph of standardized effects, cube plot, and main effects. Table S1 displays the levels and factors considered in the factorial experiment model 2k.

### 2.4. Ternary Blends Characterization

#### 2.4.1. Dynamic Mechanical Thermal Analysis

DMA studied the thermomechanical stability of the blends on a TA Instruments (New Castle, DE, USA) DMA model RSA3 in a film tension geometry. Samples were analyzed from −80 °C to 120 °C at a heating rate of 5 °C/min and a frequency of 1 Hz. The applied strain was 0.04% (strain was located in the viscoelastic linear region). Sample dimensions were 10 mm × 3 mm × 0.4 mm (*L* × *W* × *T*).

#### 2.4.2. Rheological Measurements

Rotational Rheometer model Physica MCR501 brand Anton Paar (Graz, Austria) used the geometry of parallel plates with a diameter of 25 mm. The measuring temperature was 180 °C. Master curves referenced to 180 °C were constructed from isothermal frequency sweeps obtained at six temperatures: (150 °C, 160 °C, 170 °C, 180 °C, 190 °C, and 200 °C).

#### 2.4.3. Thermogravimetric Analysis

The thermogravimetric analysis of samples employed a TGA Q600 from TA Instruments (New Castle, DE, USA). The samples were analyzed from 25 °C to 600 °C, with a heating rate of 10 °C/min. Several parameters were evaluated to characterize the thermal stability, such as *T_onset_*, maximum degradation temperature (*T_max_*), and derivative thermogravimetric scans (DTG).

#### 2.4.4. Scanning Electron Microscopy

The samples were analyzed under a scanning electron microscope, Hitachi SU3500 (Santa Clara, CA, USA). Prior to SEM analysis, cryogenically fractured surfaces from compression-molded probes were coated with gold.

## 3. Results and Discussion

The ternary mixtures were created from PLA-stiff polymer and PLA-elongated polymer, exhibiting superior mechanical properties performance [30]. In addition, polymers were selected based on an experimental design in binary mixtures (elongated or stiff) and a particular processing method (mixing time and in which the SMMA is added) for each mixture. The factors to be studied for both systems are polymer (A), concentration (B), and mixing time (C). To determine the variables significant regarding the elongation at break (*ε*_b_) and elastic modulus (*E*) parameters, the statistical analysis of the samples was carried out considering a level of significance of α = 0.05.

### 3.1. Binary Blends

#### 3.1.1. Poly(lactic acid)-Elongated Polymer

Table 2 summarizes the *ε*_b_ and *E* results, averaging values of three measurements for PLA-elongated polymer. PLA-low density polyethylene (LDPE) blends showed an increasing trend of *ε*_b_ concerning LDPE content. The 70PLA-30 LDPE/t15 blend showed an *ε*_b_ of 13.20%, corresponding to an increment of 180% compared to neat PLA/t15. When ethyl vinyl acetate (EVA) is mixed with PLA, *ε*_b_ increases because it is an elastic, flexible, and tear-resistant material [31]. Compared with neat PLA/t5, the 70PLA-30EVA/t5 and 70PLA-30EVA/t15 blends exhibit an increase in elongation percentages of 241% and 258%, respectively. The tenacity of the blends is attributed to an increase in absorbed energy and an improved ductility provided by the elastomeric material, EVA [32,33,34]. Compared with 70PLA-30EVA/t15, the *E* magnitude for 70PLA-30EVA/t5 blend decreased, particularly at a high concentration of EVA, suggesting partial compatibility concerning mixing time. Anis Sakinah et al. reported an effect of the mixing time in relation to tensile strength for EVA/waste tire dust (70/30 wt.%) owing to improved blend homogeneity [35].

Table S2 displays the factorial regression obtained after processing the response of interest, in this case *ε*_b_. The hypothesis that factors by themselves or combined [polymer (A), concentration (B), and mixing time (C)] do not influence *ε*_b_ was rejected. Thus, if a level of significance α = 0.05 is considered, the analyzed factors are statistically significant when *p* ≤ α, as in the case of A and B, correspond to the type of material used and the concentration.

To identify the factors or interactions that affect *ε*_b_, a Pareto plot of effects was constructed, Figure S1a. The graph shows that the factors which significantly influence *ε*_b_ are B and A, corresponding to the concentration and polymer used in the mixtures, respectively. Similarly, Figure S1b, where the normal chart of standardized effects indicates that the significant factors with *p* ≤ α are A and B. In the cube plot for *ε*_b_, Figure S1c, when all factors (A, B, and C) are handled at the high-level (+1), *ε*_b_ increases. Therefore, the cubic plot and the factorial regression analysis do not have a statistically significant difference on *ε*_b_, when C is handled at a low or high level (−1 or +1, 5 min or 15 min of mixing) while maintaining the other factors (A and B) at a high level (+1). The results plotted on the main effects graph for elongation are shown in Figure S1d. The increased *ε*_b_ values are generally achieved when factors A and B are under high-level conditions (+1).

Regarding parameter C, both high (+1) and low (−1) levels are considered to continue the characterization. Table S3 displays the processing conditions and polymer blends that were established from the factorial analysis. Finally, the PLA-EVA blends have enhanced properties compared to PLA-PE blends, so PLA-PE blends are discarded and the number of experiments decreases for performing the ternary mixtures.

#### 3.1.2. Poly(lactic acid)-Stiff Polymer

Table 3 summarizes *E* results, average values of three measurements for PLA-stiff polymer. The differences in *E* between poly (lactic acid)-polystyrene (PLA-PS) and poly (lactic acid)-poly (styrene-*co*-methyl methacrylate) (PLA-SMMA) blends are similar to neat PLA. Compared with neat PLA/t5, the 99 PLA-1 SMMA/t5 sample shows an *E* of 0.81 GPa, increasing 55%. The second blend, 70 PLA-30 SMMA/t5, has the highest elastic modulus value of 0.76 GPa, representing an increase in modulus of 46% to the neat PLA/t5. Based on these results, an evident influence of the type of material, concentration, and mixing time is not identified, so it is essential to use the Minitab to elucidate which factor or combination of factors can contribute directly to the stiffness of the blends. Finally, the tensile test values were used to determine the factors that influence binary blends.

The factorial regression obtained after analyzing the data for stiff blends with the variable of interest *E* is described in Table S4. Furthermore, factors A, B, and C employed are material, concentration, and mixing time, respectively. In addition, the interaction of the three analyzed factors A, B, and C was considered. The significance level utilized was α = 0.05; nevertheless, the variables directly influenced the value of *E* when *p* ≤ α.

The Pareto plot of the standardized effects for stiff blends is presented in Figure S2a. The graph shows that main effects A and C are the significant factors in the stiff blends at a 5% significance level. Likewise, it was found that the interaction between the three factors analyzed, A, B, and C, have a significant effect on the rigidity of the blend. The above is corroborated by the normal graph of standardized effects, Figure S2b, and the cube plot for *E* is shown in Figure S2c. When factors A are handled at a high level (+1) and B and C at a low level (−1), the highest elastic modulus is obtained. The identical *E* value is also obtained when factors A and B are at a high level (+1) and factor C at a low level (−1); this condition was used for the next experiments. Figure S2d shows the main effects plot for the process variables. The main effects plot shows that the type of polymer (A) and the mixing time (C) are the significant factors. It is interesting to note that factor concentration (B) at a high level (+1) and a low level (−1) has an insignificant influence on *E*, but, for this reason, both levels (or more precisely, at 1% and 30%) are considered for the following experiments. Table S5 summarizes the conditions and mixtures of the stiff blends, which will be considered for obtaining the ternary blends.

### 3.2. Ternary Blends

#### 3.2.1. Tensile Tests

The ternary blend based on (EVA)_x_-PLA_y_-(poly(S–*co*–MMA))_z_ (EPS) components were made under ideal conditions of their binary blends; specifically PLA-EVA and PLA-SMMA blends. In addition, the concentrations were 30%, 1%, and 69% for EVA, SMMA, and PLA, respectively. Furthermore, the main study focused on finding a processing method without diminishing elongation and *E* properties concerning EVA and SMMA in the PLA blend, respectively. Table 4 shows the three formulations that were elaborated employing the conditions and concentrations from selected binary blends. The first EPS 1 blend has a total mixing time of 5 min. The second EPS 2 blend had a mixing time of 15 min. Finally, the EPS 3 blend was manufactured in two steps: first, PLA was mixed with EVA for 15 min, and then SMMA was added 5 min before completing 15 min of mixing.

**Figure 1:**
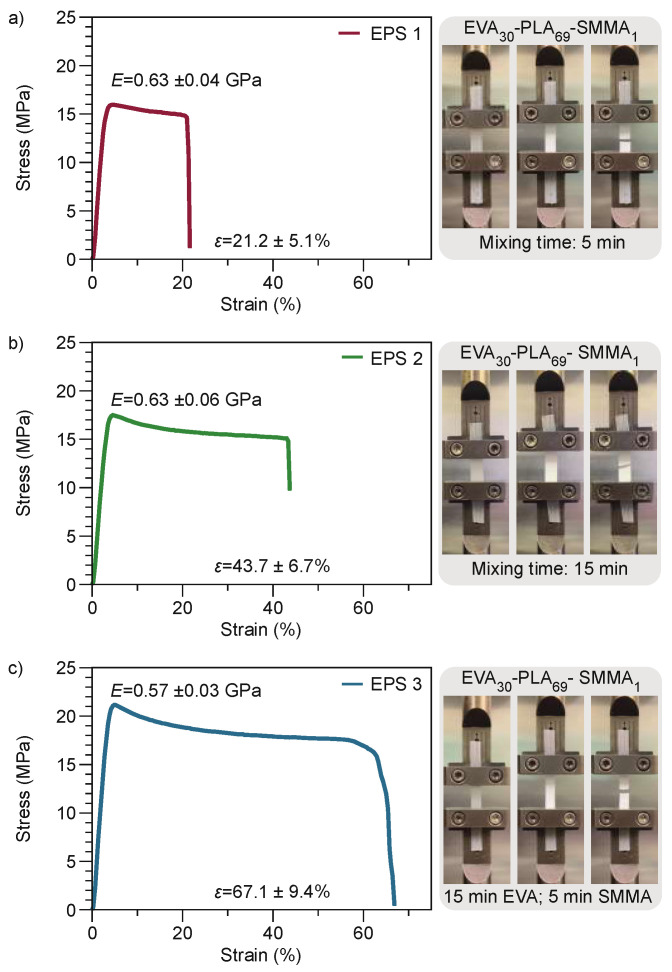
Stress vs strain curves of ternary blends. (**a**) EVA_30_-PLA_69_-SMMA_1_ (mixing time 5 min), EPS 1. (**b**) EVA_30_-PLA_69_-SMMA_1_ (mixing time 15 min), EPS 2. (**c**) EVA_30_-PLA_69_-SMMA_1_ (mixing time 15 min EVA and 5 min SMMA), EPS 3.

Figure 1 shows the elongation and elasticity modulus of the ternary blends. In addition, Table S6 exhibits the mechanical and physicochemical properties for PLA/t5, PLA/t15, and binary blends. Compared with PLA/t5, EPS 1 shows 400% and 126% increases in *ε*_b_ and *E* values, respectively. The *ε*_b_ and *E* magnitudes for EPS 1 are 21.24% and 0.63 GPa, respectively. Additionally, EPS 2 and EPS 3 blends have significant differences when compared with PLA/t15. For EPS2 and EPS3, the magnitude of *ε*_b_ increased about 931% and 1428%, respectively. The *E* value for EPS 2 and EPS 3 is 0.63 GPa and 0.57 GPa compared to PLA/t15 min with a magnitude of 0.62 GPa.

Moreover, the increase in elongation improves the ductility of PLA attributed to EVA. Nevertheless, each ternary blend has a characteristic value according to the mixing time. The results indicate that the longer the mixing time before the addition of the stiff polymer after making the PLA-EVA blend has a positive effect on elongation. Lohrasbi and Yeganeh [21] reported an *ε*_b_ of 7.3% for the PLA-EVA blend, under a processing method where the PLA was first placed, followed 2 min later by the EVA, with a mixing time of 10 min. However, when dicumyl peroxide and silica nanoparticles are added 1 min after EVA, the mixture has a super toughness effect [21]. In this study, the elastic modulus does not change substantially with mixing time. However, the mixing time for binary blends played a significant role. In accordance with the results, Zuo et al. report that the addition of SMMA only slightly affects the module elastic for PLA-SMMA-PS and PLA-SMMA-high impact PS (HIPS) [36]. A possible explanation for this could be that by adding the SMMA copolymer at 0 or 10 min, preferred placement is at the interface between the EVA and PLA. In other words, a process called compatibilization occurs, characterized by modifying the interfacial properties of an immiscible polymer blend.

#### 3.2.2. Morphology of Ternary Blends

**Figure 2:**
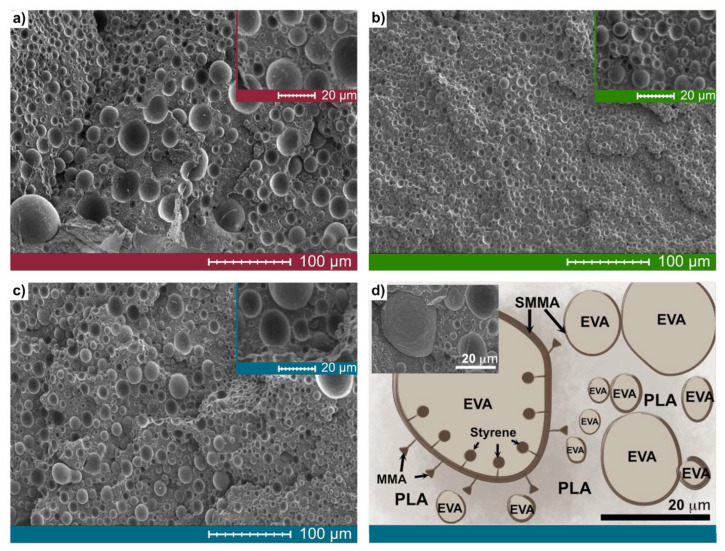
SEM micrographs of ternary blends. (**a**) EPS 1; (**b**) EPS 2; (**c**) EPS 3; (**d**) proposed model for EPS 1, EPS 2, and EPS 3 blends of all-phase coexistence.

The compatibilization process also stabilizes the morphology of the immiscible blend, PLA-EVA. For this reason, the morphology of the ternary blends was studied to confirm whether the behavior of the SMMA is as mentioned. Figure 2 shows the morphology of the ternary mixtures by SEM, as well as a representative model of ternary blend coexistence. The formation of EVA microbubbles is evident during the processing for EPS 1, EPS 2, and EPS 3, as shown in Figure 2a–c. However, there are differences in the sizes of EVA microbubbles due to mixing times and addition time (0 and 10 min) of the SMMA copolymer. Similarly, the morphology has been observed by Kugimoto et al. [37] for PLA-EVA blends, with a content of 25 wt.% vinyl acetate. Compared with EPS1 and EPS 3, EPS 2 exhibits a homogeneity in EVA microbubbles suggesting that longer processing time is needed as well as adding SMMA at the start of mixing, Figure 2b. When SMMA is added 10 min from the start of mixing, specifically EPS 3, the morphology of EVA is more heterogeneous due to the coalescence process. Furthermore, the dispersed bubbles are generated during the melt mixing process, which move through the polymer matrix and collide with one another to form fewer, larger droplets [38]. The effective compatibilization of binary blends (PLA-EVA) through the addition of a copolymer, particularly SMMA, reduces the size of the dispersed particles and, thus, the interfacial tension coefficient. Therefore, these findings suggest that SMMA has a typical role of compatibilization between PLA-EVA.

Additionally, Figure 2d illustrates a representative model of ternary blend coexistence. The proposed model has two phases: PLA and EVA. The role of SMMA is vital for mixing because it has a function similar to the compatibilizing agent. In addition, the monomeric units of styrene are miscible with EVA; and methyl methacrylate is miscible with PLA. Zuo et al. reported that PLA high-impact polystyrene blends utilize SMMA as a compatibilizing agent due to their higher miscibility with PLA and HIPS [36].

#### 3.2.3. Dynamic Mechanical Thermal Analysis

**Figure 3:**
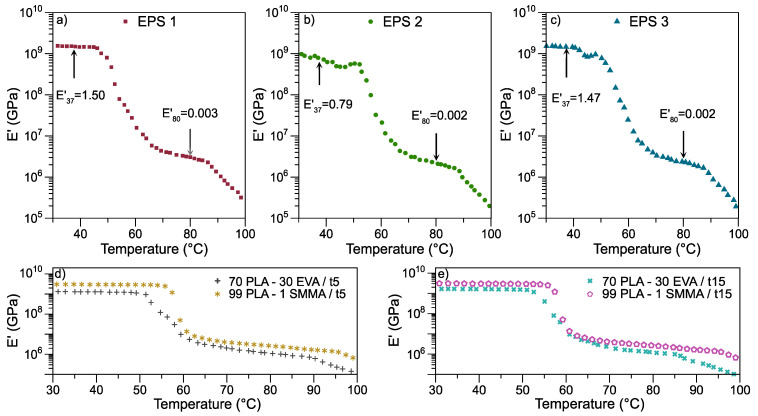
Thermomechanical behavior of ternary blends. (**a**) EPS 1, (**b**) EPS 2, (**c**) EPS 3, (**d**,**e**) binary blends.

The temperature dependence of the storage modulus (*E*′) for the selected EVA-PLA-SMMA ternary blends is illustrated in Figure 3. The DMA curves consist of three zones, including glassy state, glass transition, and rubbery state. Specifically, in the glassy zone, the *E*′ at 37 °C for EPS 1, EPS 2, and EPS 3 are 1.50 GPa, 0.79 GPa, and 1.47 GPa, respectively. Compared with EPS 1, the 70 PLA-30 EVA/t5 has a value of 1.28 GPa, suggesting that SMMA contributes to the improvement of the module. Nevertheless, at 15 min of mixing, EPS 2 and EPS3 have values similar to binary blend.

The EPS 2 blend has a substantial decrease in the *E*′ at 37 °C of 0.79 GPa, compared with EPS 1 and EPS 3. The ternary blends contain identical components and concentrations; only the mixing time and the addition times (0 and 10 min) of the SMMA copolymer are different. This factor causes a decrease in the *E*′ (37 °C) for EPS 2, suggesting that a longer mixing time and the addition of SMMA copolymer at time 0 min affects the thermomechanical property. Meanwhile, EPS 1 and EPS 3 present similar values with differences in the mixing time and the time of addition of the SMMA copolymer. However, the total mixing time of the SMMA copolymer for EPS 1 and EPS 3 are equal, i.e., 5 min.

Subsequently, *E*′ for EPS 1, EPS 2, and EPS 3 continues to decrease gradually and experiences a drastic drop at around 50 °C, which corresponds to the *T_g_* of PLA. This drastic fall can be related to the beginning of micro-Brownian movements in the polymeric chains generating the α transition; that is, there is a phase change between the glassy and the rubbery state. In this region, the presence of a peak in the loss modulus (*E*″) signal can be observed for all samples, Figure S3a–c. The maximum signal at *E*″ is associated with the maximum heat of dissipation per unit of strain [39]. Furthermore, Singla et al. [40] reported a peak height reduction of *E*″ (65 °C) owing to the polar carbonyl groups of PLA interaction with α-H of vinyl acetate in EVA. Compared with EPS 1 and EPS 3, EPS 2 has a diminish of *E*″, suggesting a significant interaction between PLA and EVA. Likewise, it is in this peak of *E*″ where the *T_g_* of the analyzed samples is estimated.

Table S7 describes the physicochemical properties, especially *T_g_* and *tan δ*, of the neat PLA, binary, and ternary blends. The miscibility of the materials, the characteristics of the interfaces, and the morphology remarkably affect the intensity and position of the peaks or dissipation signals, analyzed by DMA acquisition of the parameter *tan δ*, Figure S3d–f. The amplitude in the signals is an indication of compatibility. A slight variation in temperatures is observed in the maximum signal of *tan δ*, suggesting that slight miscibility could exist in the mixtures [39].

#### 3.2.4. Thermogravimetric Analysis

**Figure 4:**
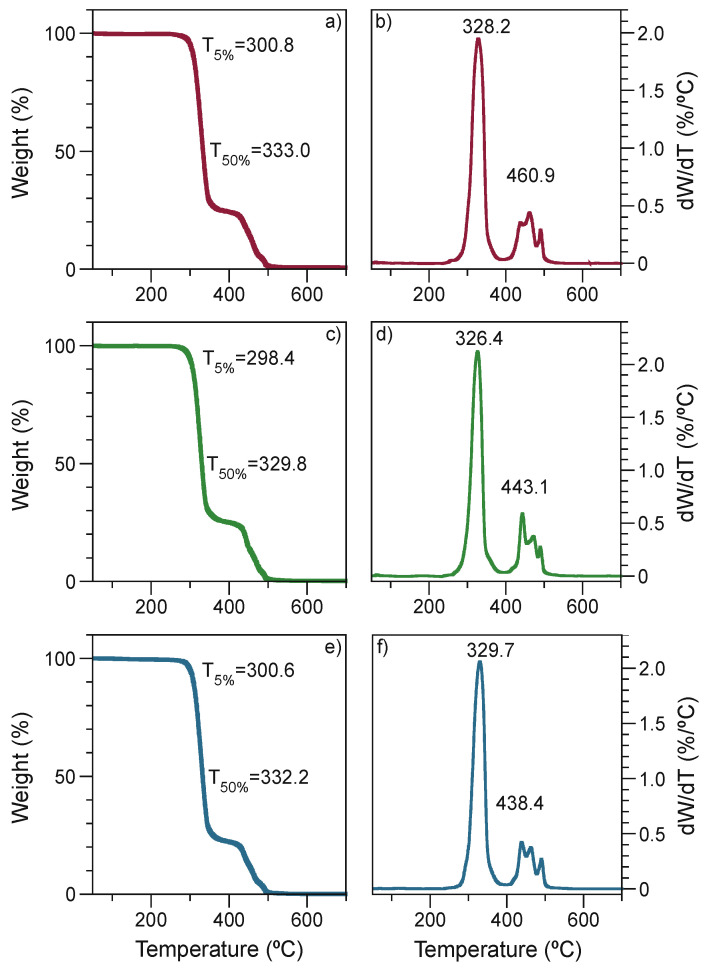
Thermograms of ternary blends. (**a**) TGA of EPS 1; (**b**) Derivative of weight loss/temperature (dW/dT) of EPS 1; (**c**) TGA of EPS 2; (**d**) dW/dT of EPS 2; (**e**) TGA of EPS 3; (**f**) dW/dT of EPS 3.

Due to the degradation suffered by the PLA during processing, it is essential to evaluate the thermal stability of polymer blends. Figure 4 shows TGA scans for EPS 1, EPS 2, and EPS 3. The degradation temperature at 5% (*T*_5%_) is around 300 °C for all ternary blends. However, there are differences. At a weight loss of 50%, EPS2 registered a diminish at *T*_50%_ with a value of 329.8 °C. Similarly, EPS 2 showed a reduction in the maximum degradation temperature (*T*_max_) close to 326.4 °C. This result can be related to the thermomechanical stability of the blends (Figure 2), where EPS 2 registers a decrease in *E’*.

The TGA parameters, including onset degradation temperature (*T*_onset_), maximum degradation temperature (*T*_max_), and final degradation temperature, for PLA, binary and ternary blends are presented in Table S7. It is clear that all ternary blends have improved thermal stability compared to PLA. EVA has two prominent peaks of degradation. Firstly, a *T*_max_ close to 344 °C, attributable to the elimination of acetic acid causing the formation of carbon-carbon double bonds, and second a degradation around 452 °C with the generation of methane, carbon dioxide, and water release due to loss of unsaturated poly(ethylene-*co*-acetylene) copolymer degradation [41,42,43]. Additionally, PLA exhibits only one degradation step with a maximum degradation of around 298 °C. Nevertheless, when EVA and SMMA are mixed with the PLA, it enhances its thermal stability, especially the EPS 3 blend.

Although EPS 2 presented a slight loss in thermal stability compared to EPS 1 and EPS 3, the formulation has a pseudo equilibrium in the particle size. Nevertheless, the thermal stability process, especially for polymer blends, depends on various factors, mainly composition and compatibility between the polymers [44]. Hence, EPS 3 and EPS 1 have improved compatibility; however, this result may be explained by the fact that the monomeric units of styrene are diffused into the EVA phase relative to the mixing time.

#### 3.2.5. Rheological Properties

**Figure 5:**
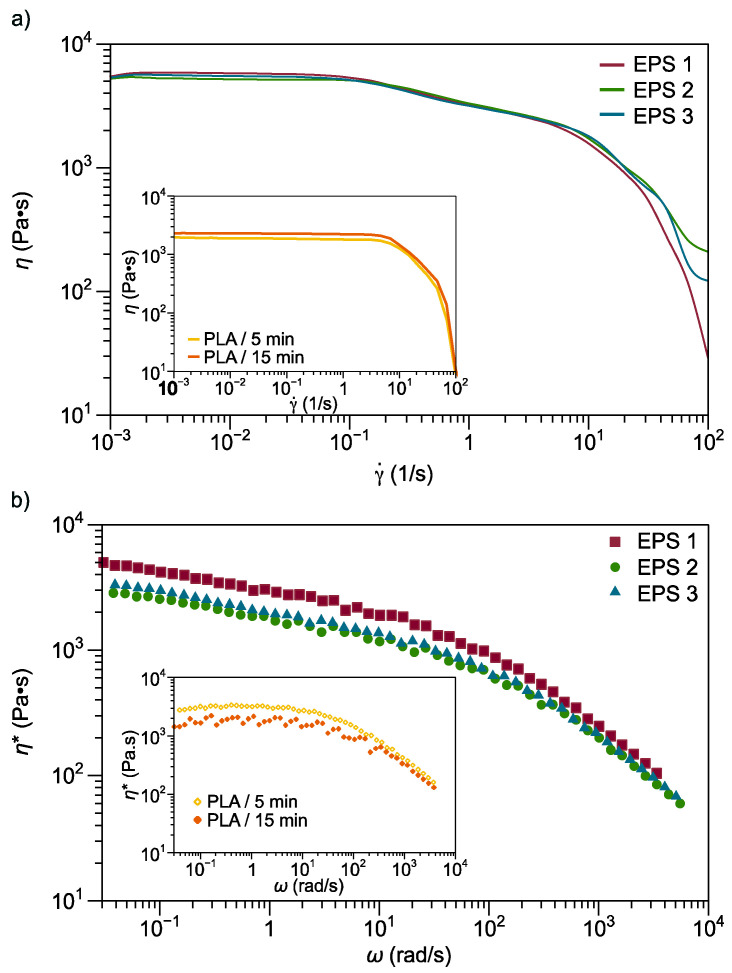
(**a**) Shear viscosity of neat PLA and ternary blends (EPS 1, EPS 2, and EPS 3). (**b**) Complex viscosity of neat PLA and ternary blends (EPS 1, EPS 2, and EPS 3).

Figure 5a shows the flow behavior of the ternary mixtures, especially EPS 1, EPS 2, and EPS 3. Compared to neat PLA 15 min, EPS 2 and EPS 3 (mixing time of 15 min) exhibit similar behavior at zero shear viscosity plateau (η_0_). Meanwhile, the EPS1 mixture experienced a decrease in viscosity at η_0,_ similarly neat PLA 5 min. The neat PLA samples present a Newtonian region at a deformation rate of about 10^1^ rad/s, and the beginning of the pseudoplastic region is presented. With the addition of the secondary components (EVA and SMMA), the Newtonian region length registers a decrease, presenting a pseudoplastic behavior at low deformation rates, around 10^−1^ rad/s.

Viscoelastic behavior is a combination of reversible elastic deformation and irreversible viscous flow due to the entanglement of the polymer chains and the slippage of the polymer chains, respectively. Compared to PLA, EVA samples exhibited marked non-Newtonian behavior because EVA has a wide distribution of relaxation time due to its polydispersity index and the existence of long-chain ramifications [37]. The previous behavior was observed by Yamaguchi et al. [45] when comparing the rheological properties of linear low-density polyethylene (LDPE) with the narrow short-chain branching distribution and low-density polyethylene (LDPE) that is characterized by long-chain branches.

Figure 5b shows the complex viscosity (η*) results for ternary mixtures and neat PLA samples at 180 °C as a function of angular frequency. Compared with PLA, EPS 1, EPS 2, and EPS 3 present a non-Newtonian behavior, decreasing the complex viscosity throughout the frequency range studied. However, the PLA has a Newtonian behavior (η* remains unchanged) in the low-frequency region, followed by a non-Newtonian behavior (reduction in η*) at high frequencies. This behavior of mixtures directly influences processability, improving efficiency, and reducing energy consumption during processing [46].

Additionally, EPS 1, EPS 2, and EPS 3 ternary blends have higher η* than the PLA samples. Particularly in the frequency range of 0.06 rad/s to 0.6 rad/s, this can be attributed to the yield stress resulting from polymer-polymer-polymer interactions [40]. The neat PLA sample (5 min) presents a diminish η* at low frequencies; however, at the frequency of 19.9 rad/s, the viscosity of this sample is similar to that recorded by the ternary mixtures. Subsequently, at a higher frequency, the values of η* of the neat PLA sample (5 min) are slightly higher, presenting similar shear thinning behavior as the ternary mixtures all profile towards a terminal region.

A disadvantage of SMMA is that it exhibits a small plateau at low frequencies with a complex viscosity close to 10,000 Pa·s at a frequency of 1 rad/s [28]. Therefore, the contribution that SMMA has in the mixture is significant because it modifies the viscosity and thus the processing conditions. For example, EPS 1, EPS 2, and EPS 3 have a η* at 0.922 rad/s of 3020, 2730, and 2950, Pa·s, respectively. Compared with ternary blends, 70PLA-30EVA/t5 and 70PLA-30EVA/t15 exhibit a value of 2160 and 1980 Pa·s [40]. Hence, there are differences at low frequencies, typical behavior of a compatibilization process [47].

Similarly, an identical behavior was observed for the viscosity flow curve. A possible explanation is that with the addition of SMMA copolymer, the viscosity of the ternary blends adjusts an increment owing to enhancement of interfacial interaction and entanglements of the PLA and EVA. However, there are differences in the magnitude of the viscosity of the ternary blend, which establishes a correlation with the mixing time and SMMA addition time. According to viscosity results, the order of magnitude from highest to lowest is EPS 1, EPS 3, and EPS 2. In other words, a higher viscosity value requires a short mixing time and adding the SMMA at time 0 min.

**Figure 6:**
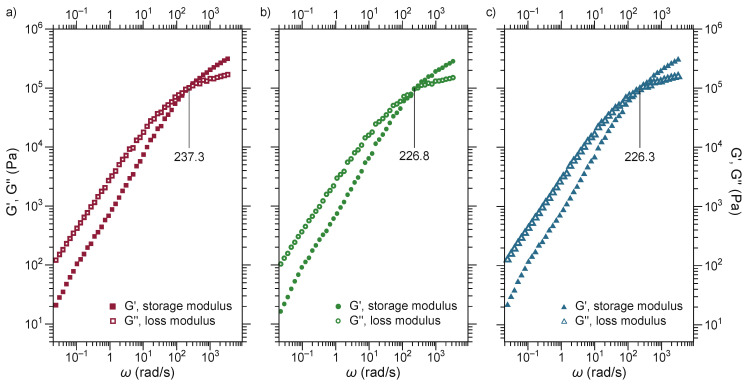
Master curves obtained at 180 °C of ternary blends. (**a**) EPS 1; (**b**) EPS 2; (**c**) EPS 3.

Figure 6 shows the master curves acquired at the 180 °C analyzing cross point when storage modulus (*G*′) is equal to the loss modulus (*G*″) representing a relaxation frequency. The crossing point of *G*′ and *G*″ is called specified frequency [48]. After this point, elastic modulus always maintained higher values than loss viscous, indicating viscoelastic nature where de-elasticity is dominant [49]. The crossover values at low frequencies involved persistence of the entanglement polymer network [50].

It is important to note that at low frequencies, the terminal zone was frequently reached with the characteristic slope of the *G*′ and *G*″, 2 and 1, respectively, when it came from homogeneous melts [51,52]. In this study, the slope values diminished at around 0.9 and 0.89 for *G*′ slope and *G*″ slope, respectively, indicating a decrease in the relaxation of the polymer chains arising due to the extent of phase interaction between PLA and EVA and SMMA polymers. Similarly, Das and Huang found identical results [51,52].

Cole–Cole curves represent the most sensitive method for analyzing multi-phase systems. These types of curves show the relationship between imaginary viscosity (η″) and dynamic viscosity (η′), and handily demonstrate the degree of miscibility of two or more components [53,54,55]. A Cole–Cole plot can be used to analyze the miscibility of polymer blends. The smooth, semi-circular shape of the plotted curves suggests good compatibility, that is, phase homogeneity in the melt. In addition, any deviation from this shape shows nonhomogeneous dispersion and phase segregation due to immiscibility [56].

**Figure 7:**
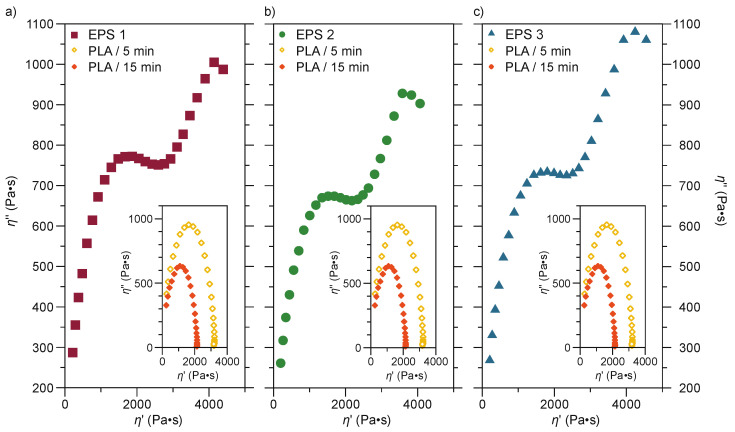
Cole-Cole plots of neat PLA and ternary blends. (**a**) EPS 1; (**b**) EPS 2; (**c**) EPS 3.

Figure 7 presents the Cole–Cole plot for the ternary blends and neat PLA. Differences between the rheological behavior of the ternary blends concerning PLA are marked. For PLA, the Cole–Cole curves resemble a semi-circle; however, the PLA sample mixed during 5 min presents a curve with a larger diameter, suggesting this sample is slightly dispersed [54]. On the other hand, ternary blends present an evident deviation from a semi-circle, corresponding to two relaxation mechanisms. In addition, the first relaxation is observed for the dominant phase, i.e., PLA, and the second semi-circle corresponds to the dispersed phase. Previous is related to two relaxation mechanisms of different morphologies, indicating coexisting phases in different internal structures such as droplet morphology or a co-continuous morphology [57,58].

Likewise, it can be observed that the second semi-circle of the ternary mixtures present in the high-frequency zone is above the arcs recorded by the neat PLA samples, suggesting an increase in viscosity and elastic energy. Although, an inverse behavior was observed by Zhao et al. [57] in mixtures of PLA and an ethylene-acrylic acid copolymer (EAA). When increasing the amount of EAA, the viscosity of the mixture decreased, indicating lower elastic energy as well as a smaller radius of the circular arcs in the high-frequency region of the Cole-Cole graphs. The notable deviation from the semi-circular shape reveals the existence of heterogeneity between the phases, which also indicates an increased viscosity in the mixtures.

**Figure 8:**
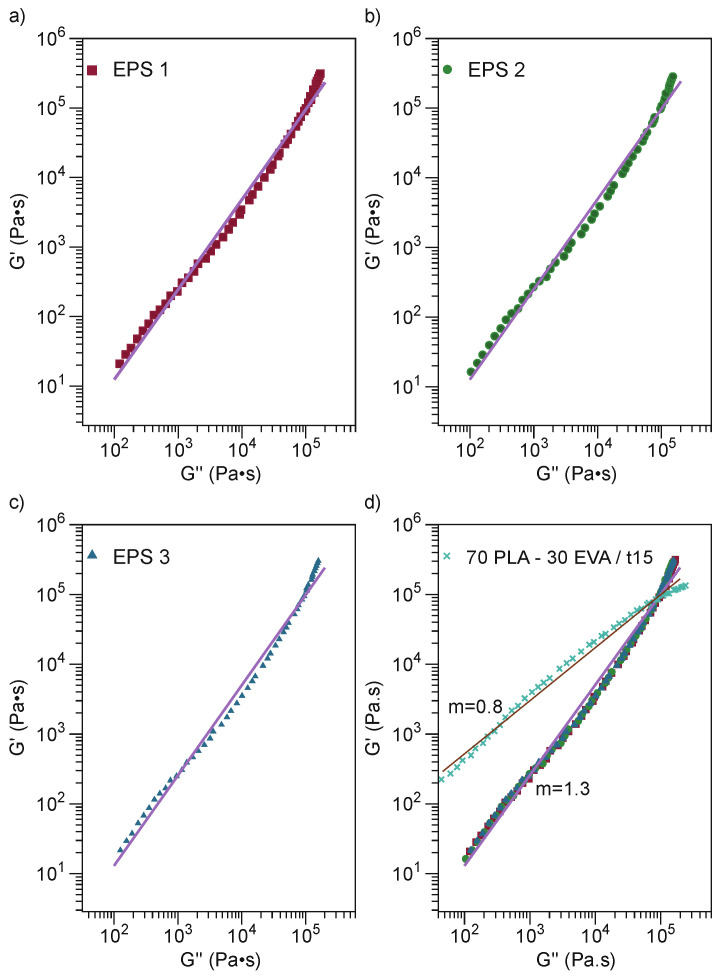
Han plots of ternary blends. (**a**) EPS 1; (**b**) EPS 2; (**c**) EPS 3; (**d**) Overall of ternary blends and 70PLA-30EVA/t15.

The Han plot has been utilized to investigate the miscibility and compatibility of the two-phase system polymer blends [54,59]. If a blend is miscible, the identical slope is observed between the blend compositions and the pure component; otherwise, it is considered an immiscible or phase-separated blend [54]. The individual components of the polymer blend have a particular viscoelastic behavior, so in a heterogeneous blend there will be different relaxation times. In addition, as a result of the variation of the slope of the Han Plot of such a fluid system, deviating gradually from 2. Figure 8 presents the Han plot of ternary blends compared with 70PLA-30EVA/t15. Figure 8a–c show EPS 1, EPS 2, and EPS 3, with an identical value for the three mixtures of 1.3. In Figure 8d, the ternary blends show an increase in relation to the binary blend. Thus, when SMMA is incorporated, it preserves the modulus of elasticity of the ternary blend yet also has an interfacial compatibilization effect due to the resulting stronger interfacial adhesion [53].

## 4. Conclusions

In the present study, ethyl vinyl acetate-poly(lactic acid)-poly(styrene-*co*-methyl methacrylate) (EPS) blends were developed. In addition, three blends (EPS 1, EPS 2, and EPS 3) with a composition of ethyl vinyl acetate (EVA) 30 wt.%, poly(lactic acid) (PLA) 69 wt.%, poly(styrene-*co*-methyl methacrylate) (SMMA) 1 wt.%, were elaborated. SMMA and EVA contributed to an overall positive effect on the stiff-elongated balance of PLA’s mechanical properties. Specifically, EVA can improve the mechanical behavior of PLA in terms of elongation at break (*ε*_b_). On the other hand, SMMA contributed to preserving the stiffness (elastic modulus, *E*) of PLA when EVA was added. In addition, SMMA is considered a handy material for improving PLA processability. Another significant role of SMMA is as a compatibilizing agent.

Moreover, the mixing and adding times of the SMMA were studied for ternary blends. The mixing time for EPS 1, EPS 2, and EPS 3 was 5 min, 15 min, and 15 min (first EVA+PLA for 10 min, plus 5 min PLA-EVA and SMMA), respectively. The order of adding polymers along with mixing times influenced the processing method, and subsequently the mechanical property, especially *ε*_b_. The EPS 3 blend featured high *ε*_b_ (67.1%), and *E* (0.57 GPa) was similar to PLA (0.6 GPa), according to dynamic mechanic analysis (DMA) results. In addition, the EPS 3 sample also has behavior analogous to EPS 2 (ε_b_ = 43.7%, *E* = 0.63 GPa); however, the elastic module is smaller compared to EPS 1 (*E* = 0.63 GPa) and EPS 2. Therefore, mixing time is essential for generating polymer blends of stiff-elongated type. Finally, the EPS 1 (*ε*_b_ = 21.2%) blend featured four times the *ε*_b_ of PLA, with 5 min of mixing and *E* nearby.

Additionally, the ternary blends were performed through the individual study of PLA stiff polymer and PLA elongated polymer blends. Furthermore, the binary blends were studied using DMA and analyzed by statistical software Minitab to determine the factors (A: type of polymer; B: concentration; C: mixing time) influencing the elongation or stiffness of the blends. The significant factors in the elongation property of the blends are the type of polymer and the concentration. By contrast, an insignificant influence was observed for the concentration regarding stiffness property, the type of polymer, and the mixing time.

## Figures and Tables

**Table 1:** Formulations of polymer blends produced.

No.	Sample	PLA Content/%	Mixing Time/min
1	neat PLA/t5	100	5
2	neat PLA/t15	100	15
3	99PLA-1LDPE/t5	99	5
4	70PLA-30LDPE/t5	70	5
5	99PLA-1LDPE/t15	99	15
6	70PLA-30 LDPE/t15	70	15
7	99PLA-1EVA/t5	99	5
8	70PLA-30EVA/t5	70	5
9	99PLA-1EVA/t15	99	15
10	70PLA-30EVA/t15	70	15
11	99PLA-1PS/t5	99	5
12	70PLA-30PS/t5	70	5
13	99PLA-1PS/t15	99	15
14	70PLA-30PS/t15	70	15
15	99PLA-1SMMA/t5	99	5
16	70PLA-30SMMA/t5	70	5
17	99PLA-1SMMA/t15	99	15
18	70PLA-30SMMA/t15	70	15

**Table 2:** Summarizes the elongation at break (*ε*_b_) and elastic modulus (*E*) results for PLA-LDPE and PLA-EVA under a mixing time of 5 and 15 min.

No.	Sample	Mixing Time/min	*ε*_b_/%	*E*/GPa
1	neat PLA/t5	5	5.30 ± 1.3	0.50 ± 0.09
2	neat PLA/t15	15	4.70 ± 0.1	0.62 ± 0.04
3	99PLA-1LDPE/t5	5	5.02 ± 1.0	0.61 ± 0.04
4	70PLA-30LDPE/t5	5	10.30 ± 2.3	0.51 ± 0.06
5	99PLA-1LDPE/t15	15	6.13 ± 1.7	0.66 ± 0.06
6	70PLA-30LDPE/t15	15	13.20 ± 6.3	0.56 ± 0.16
7	99PLA-1EVA/t5	5	8.20 ± 3.8	0.60 ± 0.19
8	70PLA-30EVA/t5	5	18.10 ± 4.8	0.29 ± 0.01
9	99PLA-1EVA/t15	15	11.90 ± 0.07	0.92 ± 0.18
10	70PLA-30EVA/t15	15	19.00 ± 8.5	0.43 ± 0.03

**Table 3:** Summarizes the elastic modulus (*E*) results for PS-PLA and SMMA-PLA under a mixing time of 5 and 15 min.

No.	Sample	Mixing Time/min	*E*/GPa
11	99 PLA-1 PS/t5	5	0.64 ± 0.05
12	70 PLA-30 PS/t5	5	0.75 ± 0.1
13	99 PLA-1 PS/t15	15	0.67 ± 0.05
14	70 PLA-30 PS/t15	15	0.57 ± 0.01
15	99 PLA-1 SMMA/t5	5	0.81 ± 0.13
16	70 PLA-30 SMMA/t5	5	0.76 ± 0.02
17	99 PLA-1 SMMA/t15	15	0.65 ± 0.05
18	70 PLA-30 SMMA/t15	15	0.69 ± 0.09

**Table 4:** Formulations of ternary blends produced.

Sample	EVA Content/%	PLA Content/%	SMMA Content/%	Mixing Time/min
EPS 1	30	69	1	5
EPS 2	30	69	1	15
EPS 3	30	69	1	* 15, 5

* First, PLA was mixed with EVA for 15 min, and then SMMA was added 5 min before completing 15 min of mixing.

## Data Availability

The data presented in this study are available in Supplementary Material.

## Supplementary Materials

The following are available online at https://www.mdpi.com/article/10.3390/polym13244279/s1. Table S1: Levels and factors considered in the factorial experiment model 2k, Table S2: Factorial regression for polymer blends with elongation capacity, Figure S1. (a) Pareto chart of standardized effects for *α* = 0.05, (b) Normal chart, (c) Cube plot, and (d) Main effects of elongation, Table S3: Conditions for formulating polymer blends with elongation capacity, Table S4: Factorial regression for stiff polymer blends, Figure S2: (a) Pareto chart of standardized effects (response elastic modulus), (b) Normal chart, (c) Cube plot, and (d) Main effects for elastic modulus, Table S5: Conditions of high stiffness blends that are considered to continue with the elaboration of ternary blends, Table S6: Mechanical and physicochemical properties of neat PLA/5 min, neat PLA/15 min, binary and ternary blends, Figure S3. Loss modulus of ternary blends. (a) EPS 1, (b) EPS 2, (c) EPS 3; and tan δ of ternary blends, (d) EPS 1, (e) EPS 2, (f) EPS 3, Table S7: TGA parameters (Tonset, Tmax, and Tf) in EVA, PLA, SMMA, and EPS ternary blends.
