# Supplementary material for: Stiff-Elongated Balance of PLA-Based Polymer Blends
Source: Polymers (Basel). 2021 Dec 7;13(24):4279. doi: 10.3390/polym13244279 (PMC8703449; doi:10.3390/polym13244279)
Supplement: Supplementary file 1 [file polymers-13-04279-s001.zip › polymers-1456695-supplementary.pdf]

polymers

MDPI

Article

Stiff-Elongated Balance of PLA-Based Polymer Blends

Mónica Elvira Mendoza-Duarte [1,2], Iván Alziri Estrada-Moreno [3], Perla Elvia García-Casillas [2,*] and Alejandro Vega-Rios [1,*]

[1] Centro de Investigación en Materiales Avanzados, S.C. Av. Miguel de Cervantes #120, Chihuahua 31136, Mexico; monica.mendoza@cimav.edu.mx
[2] Instituto de Ingeniería y Tecnología, Universidad Autónoma de Ciudad Juárez, Chihuahua 32310, Mexico
[3] CONACyT-CIMAV S.C., Av. Miguel de Cervantes #120, Chihuahua, Mexico; ivan.estrada@cimav.edu.mx
* Correspondence: pegarcia@uacj.mx (P.E.G.-C.); alejandro.vega@cimav.edu.mx (A.V.-R.)

1. Results

1.1. Binary Blends

The design of experiments was done through MINITAB-17 software. For the formulation of the binary polymer blends, a full factorial experiment model 2^k was applied. Three distinct factors, polymer, concentration, and mixing time were defined; see Table S1.

Table S1. Levels and factors considered in the factorial experiment model 2k.

		Polymer Blends with Elongation Capacity		Stiff Polymer Blends	
Factor	Description	Low Level (-1)	High Level (+1)	Low Level (-1)	High Level (+1)
A	Polymer	LDPE	EVA	PS	Poly(S-co-MMA)
B	Concentration	1%	30%	1%	30%
C	Mixing time	5 min	15 min	5 min	15 min

Table S2. Factorial regression for polymer blends with elongation capacity.

Analysis of Variance					
**Source**	**DF**	**Adj SS**	**Adj MS**	**F Value**	***p* Value**
Model	4	565.61	141.403	14.61	0.000
Linear	3	551.46	183.819	18.99	0.000
A	1	219.90	219.899	22.71	0.000
B	1	291.19	291.186	30.08	0.000
C	1	40.37	40.370	4.17	0.055
2-Way Interactions	1	14.16	14.157	1.46	0.241
A*B	1	14.16	14.157	1.46	0.241
Error	19	183.94	9.681		
Lack-of-Fit	3	15.70	5.233	0.50	0.689
Pure Error	16	168.25	10.515		
Total	23	749.56			

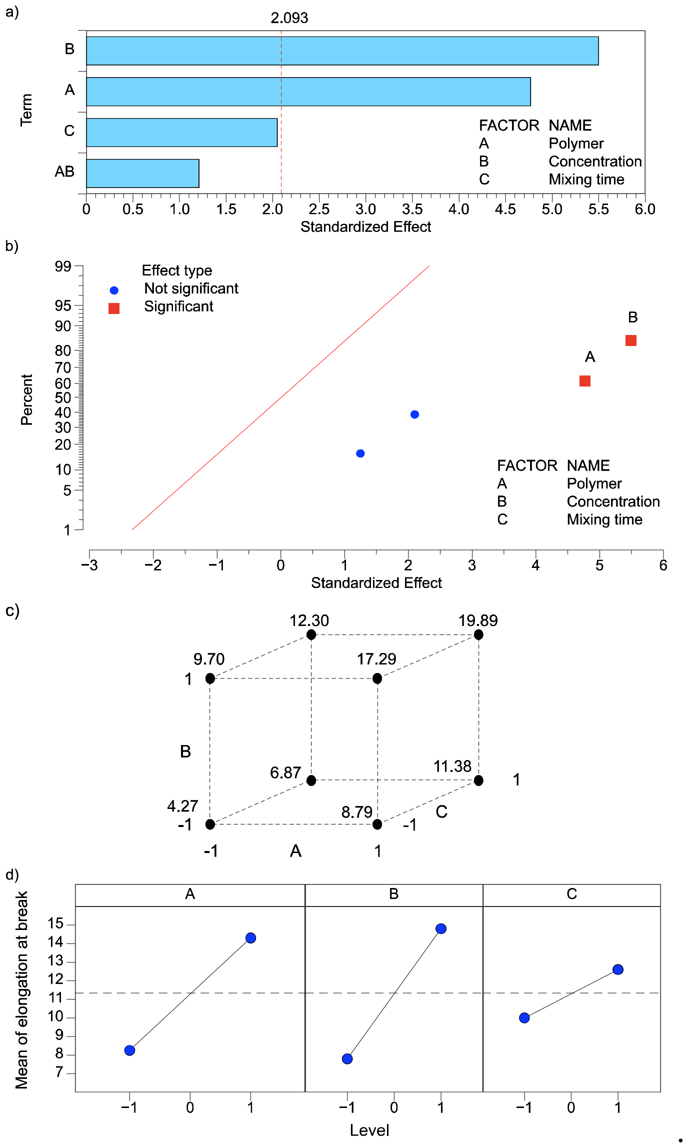

Figure S1. (**a**) Pareto chart of standardized effects for $\alpha = 0.05$, (**b**) Normal chart, (**c**) Cube plot, and (**d**) Main effects of elongation.

Table S3. Conditions for formulating polymer blends with elongation capacity.

Factor	Name	Level	Identification
A	Polymer	(+1)	EVA
B	Concentration	(+1)	30%
C	Mixing time	(−1),(+1)	5 min and 15 min

Table S4. Factorial regression for stiff polymer blends.

Analysis of Variance					
Source	DF	Adj SS	Adj MS	Valor F	Valor P
Model	7	0.126396	0.018057	3.30	0.023
Linear	3	0.084155	0.028052	5.03	0.012
A	1	0.024067	0.024067	5.26	0.036
B	1	0.000088	0.000088	0.01	0.934
C	1	0.060000	0.060000	9.81	0.006
2-Way Interactions	3	0.011568	0.003856	0.46	0.712

A*B	1	0.000400	0.000400	0.01	0.922
A*C	1	0.003174	0.003174	0.35	0.560
B*C	1	0.007993	0.007993	1.02	0.326
3-Way Interactions	1	0.030674	0.030674	6.61	0.021
A*B*C	1	0.030674	0.03674	6.61	0.021
Error	16	0.094434	0.005902		
Total	23	0.220830			

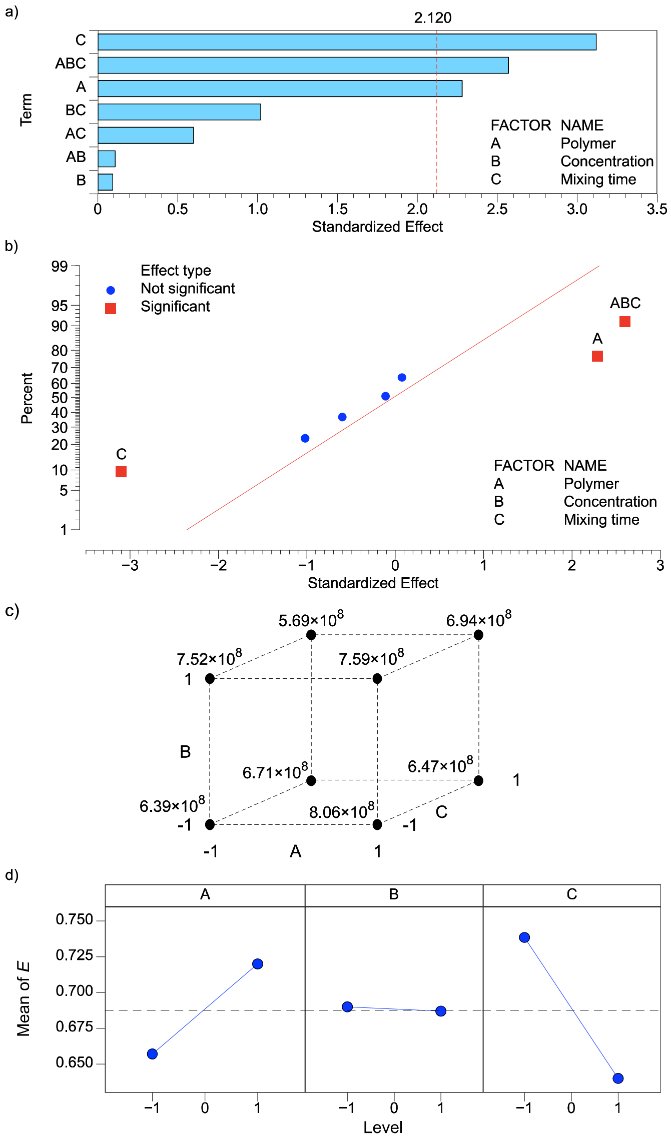

Figure S2. (**a**) Pareto chart of standardized effects (response elastic modulus), (**b**) Normal chart, (**c**) Cube plot, and (**d**) Main effects for elastic modulus.

Table S5. Conditions of high stiffness blends are considered to continue with characterization.

Factor	Name	Level	Identification
A	Polymer	(+1)	SMMA
B	Concentration	(−1),(+1)	1% and 30%
C	Mixing time	(−1)	5 min

Table S6. Mechanical and physicochemical properties of neat PLA/5 min, neat PLA/15 min, binary and ternary blends.

Sample	ε_b / %	E / GPa	E' / GPa Glassy [1]	E' / GPa Rubbery [1]	T_{g1} / °C	T_{g2} / °C	*tan δ*
**EPS 1**	**21.24 ± 5.1**	**0.63 ± 0.04**	**1.50**	**0.003**		**51.2**	**1.37**
**EPS 2**	**43.78 ± 6.7**	**0.63 ± 0.06**	**0.79**	**0.002**		**52.5**	**1.65**
**EPS 3**	**67.13 ±9.4**	**0.57 ± 0.03**	**1.47**	**0.002**		**51.6**	**1.47**
70PLA-30EVA/t5	18.1 ± 4.8	0.29 ± 0.01	1.28	0.001	−32.52	54.82	1.6
70PLA-30EVA/t15	19.0 ± 8.5	0.43 ± 0.03	1.62	0.0011	−36.41	55.07	1.56
99PLA-1SMMA/t5	-	0.81 ± 0.13	3.01	0.0025		57.4	2.1
99PLA-1SMMA/t15	-	0.65 ± 0.05	3.25	0.0027		58.2	2.1
Neat PLA/t5	5.30 ± 1.3	0.50 ± 0.09	2.85	0.002		56.3	3.05
Neat PLA/t15	4.70 ± 0.1	0.62 ± 0.04	3.01	0.002		51.6	2.69

[1] Measurement made at 37 °C and 80 °C for the glassy and rubbery zone, respectively.

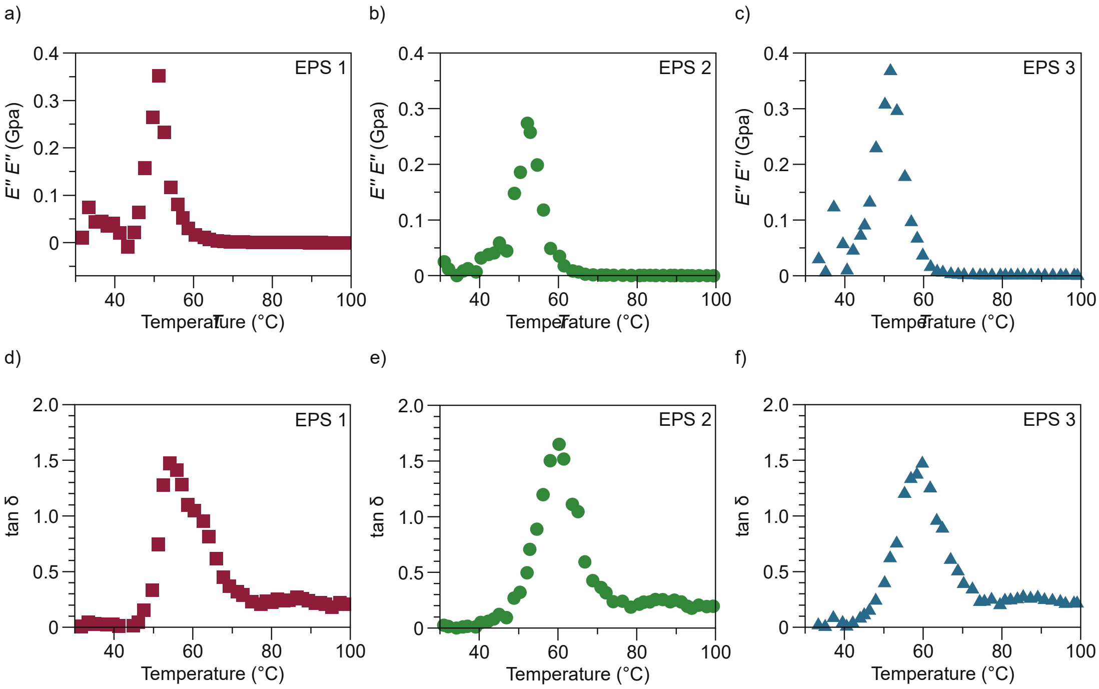

Figure S3. Loss modulus of ternary blends. (**a**) EPS 1, (**b**) EPS 2, (**c**) EPS 3; and tan δ of ternary blends, (**d**) EPS 1, (**e**) EPS 2, (**f**) EPS 3.

Table S7. TGA parameters (T_{onset},T_{max}, and T_f) in EVA, PLA, SMMA, and EPS ternary blends.

Sample	First Degradation Step (T_{d1})			Second Degradation Step (T_{d2})			Third Degradation Step (T_{d3})		
	T_{onset} /°C	T_{max} /°C	T_f/ °C	T_{onset} /°C	T_{max} /°C	T_f/ °C	T_{onset} /°C	T_{max} /°C	T_f/ °C
neat PLA / t15	223	298	253	-	-	-	-	-	-
neat EVA / t15	292	344	388	423	452	489	489	503	527
neat SMMA / t15	261	402	439	-	-	-	-	-	-
EPS 1	240	328	387	399	461	480	480	491	529
EPS 2	260	326	383	408	443	484	484	490	519
EPS 3	250	330	388	410	438	477	477	490	529
